# Defending the de dicto approach to the non-identity problem

**DOI:** 10.1007/s40592-023-00177-9

**Published:** 2023-06-26

**Authors:** Joona Räsänen

**Affiliations:** CEPDISC - Centre for the Experimental-Philosophical Study of Discrimination, Department of Political Science, Aarhus University, School of Business and Social Sciences, Aarhus, Denmark

**Keywords:** De dicto, Disability, Bioethics, Blindness, Embryo, Ethics, Harm, Non-identity problem, Reproduction

## Abstract

Is it wrong to create a blind child, for example by in vitro fertilization, if you could create a sighted child instead? Intuitively many people believe it is wrong, but this belief is difficult to justify. When there is a possibility to create and select either ‘blind’ or ‘sighted’ embryos choosing a set of ‘blind’ embryos seems to harm no-one since choosing ‘sighted’ embryos would create a different child altogether. So when the parents choose ‘blind’ embryos, they give some specific individual a life that is the only option for her. Because her life is worth living (as blind peoples’ lives are), the parents have not wronged the child by creating her. This is the reasoning behind the famous non-identity problem. I suggest that the non-identity problem is based on a misunderstanding. I claim that when choosing a ‘blind’ embryo, prospective parents harm ‘their child’, whoever she or he will be. Put another way: parents harm their child in the de dicto sense and that is morally wrong.

## Introduction

Consider the following case.

*Blind Embryo*. Wilma has a rare and strange medical condition that causes any ovum in her left ovary to produce an embryo that develops into a blind child and any ovum in her right ovary likely to produce an embryo that develops into a sighted child.^1^ Wilma has a slight preference to have a blind child so she uses in vitro fertilization to create a set of ‘blind’ embryos from her left ovum. Those ‘blind’ embryos are transferred into Wilma’s womb and 9 months later she gives birth to a blind child.

Many people believe it is wrong to choose a blind child if the alternative is to choose a sighted child. But this belief is difficult to justify. After all, if Wilma had selected a different set of embryos, she would have had a different child because the embryos had different genetic codes. The child would thus be a *different* child. This is the famous non-identity problem (Parfit 1984).^2^

Some scholars are willing to accept that parents have committed nothing wrong in such cases, because they have created a life that is worth living—as blind people’s lives are—and if they had acted differently a different child would have been born. David Boonin (2014) calls the non-identity problem the non-identity argument, indicating that there is no problem to be solved, just a valid argument to accept.^3^

In this paper, I argue that the non-identity argument, which accepts the conclusion that there is nothing wrong in creating a blind child on purpose, is based on a misunderstanding. I argue, drawing on the work of Caspar Hare (2008), that there is an important distinction, which is relevant to the topic at hand: *de dicto* betterness (worseness) and *de re* betterness (worseness). Prospective parents harm (in a de dicto sense by choosing a child who is in a worse state than the other child would have been) their child whomever she or he will be, when they create a blind child, and that is morally wrong.^4^ That is, we should be interested in the quality of life of those individuals who would exist if we acted otherwise.

Peter Singer frames the idea as follows:

…to focus only on those who exist or will exist anyway leaves out something vital to the ethics of this decision [which lives to create]. We can, and we should, compare the lives of those who will exist with the lives of those who might have existed, if we had acted differently. […W]e can and should ‘argue as it from the abyss of the non-existent’. Never having tasted ‘life’s desire’, they will ‘feel no dearth’ of life. Yet the quality of the lives they would have led is inescapably relevant to our decision. (Singer 2011, pp. 110–111)

The structure of the article is the following. In the next section, I will clarify the concepts of *de dicto* and *de re* and show, drawing on Caspar Hare, that sometimes *de dicto* harm is morally relevant. Then I will analyze David Boonin’s criticisms against Hare’s argument. I will show that *de dicto* solution to the non-identity problem can be defended.

## The de dicto solution to the non-identity problem

In this section, I argue that prospective parents wrong their child by creating and transferring a ‘blind’ embryo to the womb when they could create and transfer a different, ‘sighted’ embryo instead.^5^ The parents wrong their child in the *de dicto* sense. They thus wrong *their child*, whoever she or he will be, even though they do not wrong any genetically specific individual. My proposal therefore lines with the UK Parliament which prohibited implanting embryos known to have disabilities if other embryos with no disabilities could be implanted (Parlament of the United Kingdom 2008, p. 10).^6^

*De re* meaning can be thought of as referring to a specific person, while *de dicto* refers to anyone who happens to fit the relevant description. The general meaning of *de dicto* can, ergo, be illustrated as follows.

*Stranded Island*. I am stranded on a desert island and I launch a bottle containing a note that says, “If you find this message and bring it to my wife in New York, she will reward you with $10,000.” (Adapted from Velleman 2008, p. 237)

Here, the word ‘*you*’ refers to *whoever* finds the note. It does not matter which specific individual finds the note.

The *de dicto harm*, on the other hand can be illustrated as follows.

*Kindergarden*. A wicked misanthrope desires to blow up a schoolhouse in order to kill or mutilate the pupils. He conceals a bomb in a closet in the kindergarten room and sets a timing device to go off in six years. It goes off on schedule, killing or mutilating dozens of five-year-old children. (Adapted from Feinberg 1986, p. 154)

Here, the kids are harmed even though we do not know, at the time of concealing the bomb, which specific kids are harmed. The harm is *de dicto* harm because the harm befalls on whoever happens to be at the place at the specific time.

Caspar Hare presents the following case that is aimed to illustrate that sometimes it is precisely *de dicto* harm that is morally relevant.

*Safety Officer*. Tess is a state safety officer, whose job it is to regulate those features of the automobile that protect its occupants in the event of a collision—air bags, crumple zones, and so forth. Noticing that people in her state are not wearing safety belts, she implements some tough new regulations and, a year later, is pleased to discover evidence that they have been effective, that the severity of injuries sustained in automobile accidents has been reduced as a result of people belting up. She gives herself a pat on the back. (Adapted from Hare 2008, p. 516–517)

Hare states that one might claim to Tess that she has not done a good job. Consider someone raising the following objection.

What makes you think that you have been doing your job? Your job is to make things better for the victims of automobile accidents. But what you did made things much worse for the victims of last year’s automobile accidents. Accidents involve split-second timing. If you had just made it illegal to wear a safety belt, then most of those people would not have fumbled with the clip for five seconds before pulling out of their driveways, and, for most of them, the momentary, unhappy combination of conditions (e.g., the bicycle veering across the junction, the taxi driver rubbing his weary eyes, the crates of olive oil tottering unsteadily on the flatbed truck) that led to the accident would never have arisen. Most of them would never have been involved in accidents of any kind. (Hare 2008, p. 517)

But Hare claims, correctly, that surely Tess has done a good job. That is because Tess’ job was not to make things *de re* better for last year’s accident victims but to make it the case that last year’s accident victims were, collectively speaking, healthier than those people who would have been last year’s accident victims if she had acted otherwise. Her job was to make things *de dicto* better for the accident victims: better for *whoever* the victims turned out to be.

Now, Hare’s *Safety Officer* is analogous to *Blind Embryo*. In *Safety Officer* Tess has a special role that makes it appropriate for her to be partial to a certain group of people (accident victims) and to have her special concern for the health of that group of people guide her behavior. Likewise, in *Blind Embryo*, Wilma has a special role that makes it appropriate for her to be partial to a certain group of people (her offspring) and to have her special concern for the health of that group of people guide her behavior.^7^

But Boonin objects to Hare’s solution because apparently, *Safety Officer* is itself open to two possible interpretations about the *de dicto* obligations.^8^ As Boonin states:

[T]he fact that Tess’s claim that “my job is to make things *de dicto* better for the accident victims” can itself be understood in two ways. On one interpretation, the claim that the *de dicto* sense is relevant in the Tess case is plausible, but on that interpretation, Wilma’s act of [creating a blind child] does not make her child *de dicto* worse off. On the other interpretation, Wilma’s act of [creating a blind child] does make her child *de dicto* worse off, but on that interpretation, it is not plausible to say that the *de dicto* sense is relevant in the Tess case. The case of Tess thus fails to identify a morally relevant sense in which (…) the non-identity argument is false. (Adapted from Boonin 2014, p. 34)

So, according to Boonin, in *Safety Officer* Tess’s job is either to.

(A) make sure that the person (S) who is in the accident suffers less harm than would have been suffered by the person (S’) who would otherwise have been in the accident. *or* Tess’s job is to

(B) make sure that the person (S) who is in the accident ends up having a higher level of health after the accident than the person (S’) who would otherwise have been in the accident would have ended up having.

Boonin claims that in the *Safety Officer*, the correct policy is to make sure that whoever happens to be in the accident suffers less harm than would have been suffered by the person who would otherwise have been in the accident (A). According to Boonin, it seems implausible that Tess, as a safety officer, would do a good job simply by redirecting accidents towards healthier people (B).

Because in the *Safety Officer* the correct policy is to reduce the severity of the accidents rather than directing the accidents towards healthier people, according to Boonin (and Purves 2013), Hare’s *Safety Officer* fails to be on par with other non-identity scenarios such as the *Blind Embryo*. Thus Hare’s distinction between *de re* and *de dicto* harms does not solve the non-identity problem.

It is true that Tess should be concerned about the severity of harm rather than the amount of health the accident victim would have after the accident. But that is because *Safety Officer* fails to be on a par with *Blind Embryo,* or with other procreative cases. In *Safety Officer*, Tess does not know—and cannot know—the state of health of possible accident victims before they have the accident. Therefore, Tess’ only reasonable option for guiding her behavior is to be concerned with the amount of harm the accident itself causes—not the amount of health victims will have after the accident.

On the other hand, in *Blind Embryo* Wilma can reasonably expect what the health of her possible children will be like. Her child would be either blind or sighted.^9^ Now, consider a thought experiment that I believe is more akin to *Blind Embryo* than Hare’s *Safety Officer*.

*Infected Water.* Lisa is a water inspector and her job is to supervise the water distribution to the city. One day, when the safety features of the water supply are temporarily disabled, she finds out that water contains a nasty bacterium, which infects some of the people who drink it and causes them to have severe pain and discomfort. Lisa has two options. She could direct water to Suburbicon, an area of healthy and wealthy people. Call residents of Suburbicon *strong*. Or she could direct water to Shacktown, an area of the sick and poor. Call residents of Shacktown *weak*. If Lisa directs water to Suburbicon many but not every resident of Suburbicon get sick and their health is reduced. If Lisa directs water to Shacktown many but not every resident of Shacktown get sick and their health is reduced. If Lisa does nothing, the pressure in the water tank causes the infected water to spread all over the town (because of the disabled safety features) eventually infecting everybody in the Suburbicon and Shacktown! Assuming that the *strong* would lose more health and suffer more pain and discomfort than the *weak*, and assuming that the *weak* are already in less health than the *strong* are, what should Lisa choose?

To illustrate Lisa’s options, see the following:

**Table:** 

Option 1	Option 2
Channel water to Suburbicon	Channel water to Shacktown
Some of the strong get sick;	Strong do not get sick;
Reduce individual health 100—> 90	Individual health stays at 100
Weak do not get sick;	Some of the weak get sick;
Individual health stays at 80	Reduce individual health 80—> 75

I suggest that Option 1 is morally preferable. That is because when choosing option 1, Lisa is protecting the weakest of the possible victims, even when the amount of pain and discomfort would be reduced more by choosing option 2 (in option 1 the loss of individual health is 10 units, in option 2 the loss of individual health is 5 units). It would be especially cruel and vile for Lisa to direct the harm against the people who are already in a weak position and not as capable of receiving the harms as others. Lisa’s job (or a moral obligation) is therefore to ensure that whatever person turns out to occupy the role of the accident victim ends up as healthy as possible. Therefore, Lisa should choose policy 1 because in that case the accident victim will end up having a health score of 90, and that is better than choosing policy 2, where the accident victim would end up having a health score of 75.

Lisa has an obligation to *de dicto* betterness, even though she will make things *de re* worse for those who drink the water because of her actions and who would not drink the water if she would act differently. While *Infected Water* is not exactly like *Blind Embryo*, it is analogously closer to *Blind Embryo* than *State Officer* because unlike *State Officer*, in *Infected Water* and *Blind Embryo* it is known how healthy the possible victims are. To put it another way: Lisa knows the health of people in different parts of the city and Wilma knows the health of her child (whether the child will be sighted or blind). Wilma, like Lisa, should be concerned about the weakest and most vulnerable. Since Lisa should ensure that whoever person turns out to occupy the role of the accident victim ends us as healthy as possible, so does Wilma. Wilma has, therefore, a morally relevant obligation to the *de dicto* betterness of *her child*. So Wilma should select the embryo that will produce a sighted child.

Thus non-identity problem can be solved, or bypassed, with *de dicto* betterness, at least in this particular scenario. Next, I will consider some objections against the proposed *de dicto* solution.

## Objections

Boonin argues that if it is correct that we should ensure that whatever person turns out to occupy the role of the child ends up as healthy as possible, then the implications are even more difficult to accept than accepting the conclusion of the non-identity argument. Consider the following case raised by Boonin.

*Medical Doctor.* I have just received my license to practice medicine and am in the process of establishing my first practice. At the moment, I have no patients, but because the town I have moved to has long suffered from a shortage of doctors, I am immediately inundated with requests from people who would like me to be their doctor. In fact, I have twice as many applications as I can accept and am trying to decide which of the applicants to take on as patients. (…) if Hare’s explanation of the source of Tess’s obligations is correct, then I am morally obligated to decide which people to take on as patients by appealing to *de dicto* considerations. (…) I should be guided by the *de dicto* concern for what would be best for the health of “my patients.” And this means that I must choose the healthier people to accept as patients. (Adapted from Boonin 2014 at. p. 37–38).

So, according to Boonin, if what matters morally is the amount of health the accident victims will have after the accident, and not the amount of damage that is prevented when choosing who suffers the accident, then in cases like *Medical Doctor*, we should be concerned with what is the total health of the victims after the accident.

If my interpretation of Hare’s *de dicto* obligations is correct and *Infected Water* case is analogous to *Blind Embryo* then the doctor has an obligation to choose the healthiest people as his patients. That is because, if Wilma should ensure that whatever person turns out to occupy the role of her child ends up as healthy as possible then it seems that the doctor should ensure that whatever person turns out to occupy the role of his patient ends up as healthy as possible—which means that the doctor should select the healthiest people to be his patients. But that cannot be right.

It seems that there is a puzzle. In some cases, like *Infected Water*, there is a *de dicto* obligation to make sure that whatever person turns out to occupy the role of S *ends up as healthy as possible*, yet in other cases, like in *Medical Doctor*, it seems that the obligation is to make sure the person occupying the role of *S ends up suffering less harm* than someone else otherwise would. The problem is this: how can we know which of these examples (*Infected Water* or *Medical Doctor*) should serve as a guide to our behavior for procreation cases like *Blind Embryo*?

The solution can be found, I believe, from the wider asymmetry: the asymmetry of causing people to exist. There is a widely-held belief that there is a moral reason not to cause a person to exist if her life would be miserable, but no moral reason to cause a person to exist because his life would be worth living. Jeff McMahan frames this asymmetry in the following way.

[T]he expectation that a person would have a life worth living does not by itself provide a moral reason to cause that person to exist. […yet] the expectation that a person would have a life in which the intrinsically bad elements outweigh the good does provide a moral reason not to cause that person to exist. (McMahan 2013, p. 15–16)

McMahan calls this the *Procreative Asymmetry*. In procreation cases (such as in *Blind Embryo*), we are—intuitively—more concerned about *not to harm* than *to benefit*, as the Procreative Asymmetry shows. In *Infected Water,* Lisa is concerned with about the *harm* and which people she should protect from it. She is not benefitting the victims; she is protecting them from harm.^10^ But in *Medical Doctor* the doctor is concerned about the *benefit* and which people he should benefit. He is not protecting victims from harm; he is benefitting them by curing and giving them medical assistance.

Because *Infected Water* is protecting-from-harm case, while *Medical Doctor* is benefitting case we should look for guidance from *Infected Water,* when we want to find out what is the correct policy in *Blind Embryo*. The *Infected Water* (rather than *Medical Doctor*) thus is more akin to *Blind Embryo* and other cases of procreation where we have the intuition of *Procreative Asymmetry*. Therefore, *Medical Doctor* fails to be a convincing counterexample against the *de dicto* solution to the non-identity problem.

At this point, one might object by claiming that in *Medical Doctor* the doctor is not offering his patients *pure benefits*. Instead of offering pure benefits, when the doctor cures diseases he is preventing harm or preventing further harm. Thus, the analogy breaks down and the intuitions behind procreative asymmetry cannot be used as evidence anymore.^11^

However, if one believes there is a morally relevant category of pure benefits, one should still be persuaded by the argument presented in this paper*.* In relation to pure benefits, Seana Shiffrin (1999) makes two important points. First, pure benefits are benefits that are just goods and which are not also removals from or preventions of harm. Second, there is a substantial asymmetry between the moral significance of harm delivered in avoiding substantial, greater harms and harms delivered to bestow pure benefits. According to Shiffrin, it is much harder to justify harm when bestowed pure benefits than to justify harm to prevent greater harm.

Suppose one believes that all this is correct. If causing someone to exist is a benefit it seems to be a pure benefit. Bringing a person into existence does not prevent that person from more severe harm—it offers a benefit that is good itself.^12^ But it also necessarily harms the person (Benatar 2006). Since bringing a person into existence harms the person while offering pure benefits (instead of preventing greater harms) it is in a serious need of justification.^13^ If bringing someone into existence is in a serious need of justification—even when one chooses ‘best possible children’,^14^ then surely bringing a less optimal children (such as blind children) into existence needs even more serious justification: so at least a somewhat similar conclusion (to the one in this paper) has been reached—albeit from a different path.

One might also object against the argument presented in this paper by raising the following case.

*Car Seat*. Suppose you are a parent shopping for a car seat for your child. You have narrowed it down to two options, both of which are equally safe. There is a cheaper car seat that will be less comfortable for your child, and there is an expensive car seat that will be more comfortable. You opt for the cheaper car seat, so that you can spend the money you save on a fancy dinner. (Haramia 2014: 72).^15^

Now, if the de dicto betterness is what matters morally, then the parents should choose the more expensive car seat because when doing so they make their child slightly better off. However, many people find it difficult to believe that the parents would do something morally wrong when choosing the cheaper but equally safe car seat.

I would be inclined to think that while the parents have a moral *reason* to choose the more expensive car seat (because it makes their child better off), they are not morally *obligated* to do so, because the benefit for the child is not that significant. Now, suppose that instead of choosing to create a ‘blind’ embryo or a ‘sighted’ embryo the parents would choose between an embryo that would likely produce a child with slightly better than average vision or a child with slightly worse than average vision, again, it seems that while there is a moral reason to choose the better off child (the one with better vision), it would not be seriously immoral to choose the other one either. So there isn’t a qualitative difference between this case and the case where the parents choose blind or sighted embryo—only a quantitative one.^16^ But losing sight altogether makes the child much worse off (in de dicto sense) and so is a major disadvantage. Because of this, I would say that there is a moral obligation not to create and select the ‘blind’ embryo.

Someone might question whether the argument presented in this paper is just another way to reach anti-natalism.^17^ While I have certain sympathies towards anti-natalism (Räsänen 2021, 2023), I remain skeptical whether the argument presented in this paper is anti-natalist. I have argued that the parents wrong their child if they harm her in the de dicto sense and they do this when they do not choose the best possible child out of a range of possible children they might have. But most cases of reproduction are not like *Blind Embryo* where the parents know whether the child will have a certain disease, disability, or a feature. Thus, in many cases where the prospective parents are unaware of the genetic features of their children or when the possible children do not differ regarding their genetic features, the parents do not wrong their children by bringing them into existence (at least not in the way de dicto argument suggests). For instance, if Wilma’s ovaries generate only ova which produces blind children, she does not wrong her child by procreating a blind child because there would be no other possible children who could be better off in a de dicto sense than the blind child she is having.

## Conclusion

I have argued here that the non-identity problem is based on misunderstanding. There is a misunderstanding because sometimes we harm people in *de dicto* sense, even though we do not harm them in *de re* sense. I defended the *de dicto* solution to the non-identity argument against the criticism by David Boonin. I argued that the prospective parents harm their child, whoever he or she will be, if they select a blind child. So even though our life choices cause different people to exist than if we had chosen otherwise, we should consider what would be the quality of life of those that would have existed (but do not exist) if we had acted otherwise.^18^

## Footnotes

1 Such a medical condition does not exist. I have made it up solely to introduce the problem.

2 Derek Parfit originally introduced the problem by using an example of a 14-year-old child who could either have a child now or wait and have a child later when she is more mature. In *Reasons and Persons* (and again in volume 2 of *On What Matters*), Parfit himself rejected, though in different terms, a somewhat similar solution to the non-identity problem I am defending here. However, later, in a posthumous publication (2017), Parfit defends a related view: ‘a wide person-affecting principle’, which is somewhat close to the solution defended in this paper. I thank an anonymous reviewer at *Bioethics* for bringing Parfit’s posthumous publication to my attention. Chelsea Haramia (2013a) has also defended the de dicto solution to the non-identity problem. I thank an anonymous reviewer at *Medicine, Health Care and Philosophy* for bringing Haramia’s article to my attention. While the conclusion I aim at this paper is not entirely new, for instance, it also bears some resemblance to Kumar (2018), I believe the way I argue for it is a novel one.

3 In a recent work, Cristian Puga-Gonzales (2019) also seems to accept the conclusion by rejecting the principle what he calls the Prohibiting the Implantation of Deaf Embryos. Technically the principle should be Prohibiting the *Transfer* of Deaf Embryos (or in the case of this paper: blind embryos), because it is not (yet) possible to implant embryos to the womb. The embryos are technically transferred to the womb with a hope, but no guarantee, that it leads to implantation of some of the embryos. I thank Daniela Cutas for this remark. The possibility of more than one embryos to implant raises interesting philosophical problems (see. Räsänen 2022).

4 There are many different accounts of harms. For the purpose of this paper, I rely on an intuitively compelling account of harm: the counterfactual comparative account (CCA) of harm. According to it, an event is harmful when it makes someone (or something) worse off than s/he (or it) would have been had that event not occurred. For more about CCA see Klocksiem (2012).

5 Of course, the embryos themselves are neither hearing nor blind. When talking about blind or sighted embryos, I am talking about embryos that are likely to produce either a blind or a sighted child.

6 For discussions on the morality of creating a deaf child on purpose, see McDougall (2007) and Wallis (2020). More general arguments regarding parents' responsibilities when selecting for children with certain traits see Sparrow (2013) and Haramia (2013b).

7 For a defence of Hare’s de dicto solution see also Haramia (2013a, b).

8 For a different criticism against Hare, see Weinberg (2013).

9 This should not be understood implying that all that matters for an individual health is the ability to see. All we know here is that these embryos are healthier, in *a certain respect*, than others and we assume the embryos would otherwise produce children with similar health. I thank an anonymous reviewer at *Monash Bioethics Review* to urge me to clarify this point.

10 One might claim that Lisa is not protecting victims from harm because anyone who is harmed if she chooses one particular route for the water, could have *not* been harmed if she had chosen the other option. Thus she is always harming (or allowing harm to) the one who drinks the infected water. While it is true that all victims are harmed in *de re* sense, before Lisa makes her choice where to channel the water, she is guided by the *de dicto* concern for what would be best for the health of the victim: she protects the potential victim with her actions by making sure he or she never ends up being a victim because of her actions in the first place.

11 I thank an anonymous reviewer at *Bioethics* for raising this objection.

12 See for example Jacobs (2015).

13 Benatar’s antinatalism is, of course, highly controversial and many reject it.

14 See Savulescu (2001) for why we should, allegedly, choose the best possible children.

15 This objection, that is found in Haramia (2014) and is credited to David Boonin, was raised to me by an anonymous reviewer at *Medicine, Health Care and Philosophy*.

16 I do not argue here at what point this moral reason becomes a moral obligation since I do not think that is relevant for the purpose of this paper. What is relevant is that there is always a moral reason (sometimes very minor) to choose the better off child and I believe it is a reasonable assumption that when the child is prevented to have a vision at all, he or she is significantly worse off (in de dicto sense) so that it is morally obligatory to choose the better off sighted child. The view presented in this paper might imply *Procreative Beneficence* (Savulescu 2001) and the conclusion that if you must make babies there is a reason to make the best possible babies (Häyry 2004). I think these are reasonable conclusions.

17 For instance, an anonymous reviewer at *Medicine, Health Care and Philosophy* suspects this.

18 The argument presented here probably does not solve the non-identity problem in all cases but that has not been my intention in the first place.
